# Superoxide dismutating molecules rescue the toxic effects of *PINK1* and *parkin* loss

**DOI:** 10.1093/hmg/ddy069

**Published:** 2018-02-24

**Authors:** Alice Biosa, Alvaro Sanchez-Martinez, Roberta Filograna, Ana Terriente-Felix, Sarah M Alam, Mariano Beltramini, Luigi Bubacco, Marco Bisaglia, Alexander J Whitworth

**Affiliations:** 1 Molecular Physiology and Biophysics Unit, Department of Biology, University of Padova, 35131 Padova, Italy; 2 MRC Mitochondrial Biology Unit, University of Cambridge, Cambridge Biomedical Campus, Cambridge CB2 0XY, UK

## Abstract

Reactive oxygen species exert important functions in regulating several cellular signalling pathways. However, an excessive accumulation of reactive oxygen species can perturb the redox homeostasis leading to oxidative stress, a condition which has been associated to many neurodegenerative disorders. Accordingly, alterations in the redox state of cells and mitochondrial homeostasis are established hallmarks in both familial and sporadic Parkinson’s disease cases. *PINK1* and *Parkin* are two genes which account for a large fraction of autosomal recessive early-onset forms of Parkinson’s disease and are now firmly associated to both mitochondria and redox homeostasis. In this study we explored the hypothesis that superoxide anions participate in the generation of the *Parkin* and *PINK1* associated phenotypic effect by testing the capacity of endogenous and exogenous superoxide dismutating molecules to rescue the toxic effects induced by loss of *PINK1* or *Parkin*, in both cellular and fly models. Our results demonstrate the positive effect of an increased level of superoxide dismutase proteins on the pathological phenotypes, both *in vitro* and *in vivo*. A more pronounced effectiveness for mitochondrial SOD2 activity points to the superoxide radicals generated in the mitochondrial matrix as the prime suspect in the definition of the observed phenotypes. Moreover, we also demonstrate the efficacy of a SOD-mimetic compound, M40403, to partially ameliorate *PINK1/Parkin* phenotypes *in vitro* and *in vivo.* These results support the further exploration of SOD-mimetic compounds as a therapeutic strategy against Parkinson’s disease.

## Introduction

Parkinson’s disease (PD) is a neurodegenerative motor disorder associated with the preferential death of dopaminergic neurons in the *Substantia Nigra pars compacta*. Even though the etiology of this disease, which includes environmental and genetic causes, is still not completely elucidated, oxidative stress and mitochondrial dysfunction have been identified as major factors involved in the pathogenesis of PD. In fact, alterations in the redox cellular state and mitochondrial homeostasis are common hallmarks in both familial and sporadic PD cases (1).

Mitochondria are dynamic organelles, organized in an interconnected network, with the ability to fuse and divide. The fusion and fission pathways are implicated in homeostatic maintenance of mitochondrial DNA stability and respiratory function, as well as influencing apoptosis (2). As a consequence, the process known as mitochondrial quality control, which refers to mitophagy, organelle fission, fusion and subcellular translocation, is essential for mitochondria performance. The malfunctioning of mitochondrial dynamics results in the accumulation of defective organelles, leading to oxidative stress and cell death (3). On the other hand, even oxidative stress can affect mitochondrial morphology and functions. Accordingly, a growing body of evidence suggests that redox cellular state and mitochondrial homeostasis are tightly interconnected and that alterations in reactive oxygen species (ROS) levels go in parallel with changes in mitochondrial dynamics (3).

Mutations and deletions in the genes *Parkin* and *PINK1*, accounting for the majority of autosomal recessive early-onset forms of PD (4), have been demonstrated to affect both mitochondrial and redox homeostasis (5). Remarkably, the first definition of the function played by the proteins encoded by these genes at the mitochondrial level came from studies carried out in *Pink1* and *parkin* knockout *Drosophila* models (reviewed in (6)). In addition to mitochondria alterations, *PINK1* or *Parkin* deficiency has also been reported to be associated with ROS production and/or increased susceptibility to oxidative conditions: *Pink1* deficient flies show increased sensitivity to multiple insults including oxidative stress (7), while *parkin* mutant flies are characterized by an alteration in oxidative stress response (8) and an increased sensitivity to oxygen radical injury (9). With the exception of muscular defects, phenotypes similar to those firstly described in *Drosophila* were subsequently described in other *PINK1* and *parkin* knock-out cellular and animal models (10–15).

In light of the strong evidence supporting a connection between mitochondrial redox homeostasis and mitochondria dynamics (3), an attractive hypothesis is that the handling of mitochondria fragmentation through endogenous or exogenous antioxidants could be a valid therapeutic strategy to combat PD. In this frame, superoxide anions, mainly produced during mitochondrial oxidative phosphorylation (16), appear to be the most suitable targets for antioxidant therapies. Superoxide radicals are considered the ‘primary’ ROS as they can further react with other molecules to produce more reactive ‘secondary’ ROS, such as hydroxyl radicals and peroxynitrites (3). Recently, we have demonstrated the protective role of the superoxide dismutase enzymes, SOD1 and SOD2, and of the SOD-mimetic agent M40403 in paraquat-based cell and fly models of PD (17). In light of these results, the aim of the present work was to verify whether endogenous and exogenous superoxide dismutating molecules might rescue the toxic effects induced by *PINK1* and *Parkin* loss in both cellular and fly models. Our results indicate that boosting either endogenous or exogenous SOD capability can ameliorate pathologically linked phenotypes *in vitro* and *in vivo*. Moreover, our results indicate a more prominent detrimental effect coming from mitochondrial superoxide. These results support and expand the field of the potential use of SOD-mimetic compounds as a therapeutic strategy in Parkinson’s disease.

## Results

### *PINK1* and *Parkin* mutagenesis using the CRISPR/Cas9 system

**Figure 1.**
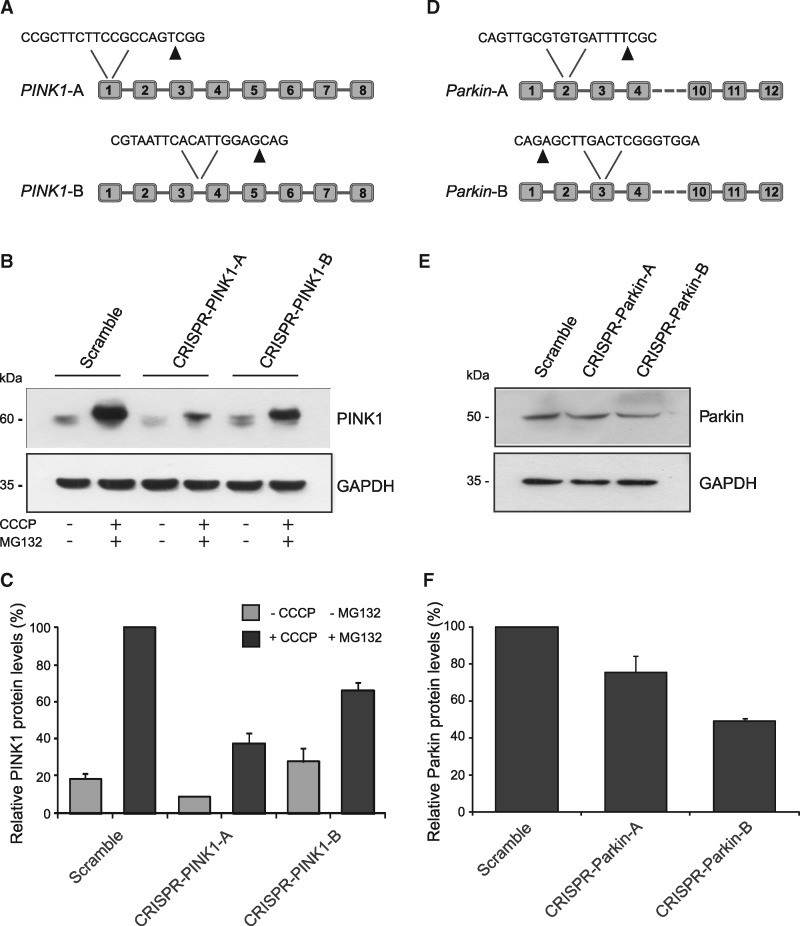
*PINK1*- and *Parkin*-editing in HeLa and HEK-293T cells. (**A**) Schematic representation of the gRNA target sequences in *PINK1* gene. Arrows indicate Cas9 DNA cutting sites. (**B**) Western blot analysis of PINK1 in HeLa cells transiently expressing scramble, CRISPR-PINK1-A or CRISPR-PINK1-B plasmids. GAPDH signal was used as loading control. (**C**) Densitometric analysis of PINK1 levels normalized to GAPDH expressed in percentage. Data are expressed as mean ± SEM of three independent experiments. (**D**) Schematic representation of the gRNA target sequences in *parkin* gene. Arrows indicate Cas9 DNA cutting sites. (**E**) Western blot analysis of Parkin in HEK-293T cells transiently expressing scramble, CRISPR-Parkin-A or CRISPR-Parkin-B plasmids. GAPDH signal was used as loading control. (**F**) Densitometric analysis of Parkin levels normalized to GAPDH expressed in percentage. Data are expressed as mean ± SEM of three independent experiments.

To generate *PINK1* and *Parkin* deficient cells, we used the CRISPR/Cas9 technology to modify genomic DNA in an irreversible manner (18). The CRISPR/Cas9 adaptive approach uses a single guide RNA (sgRNA) to direct the Cas9 nuclease and specifically generate double strand DNA breaks, resulting in random insertions and deletions in the specific genomic site, via error-prone non-homologous end joining repair mechanisms (18). Two different target sequences were designed for each gene for CRISPR/Cas9 mediated mutagenesis (Fig. 1). These sequences were cloned in an expression vector containing CD4 as a reporter gene to label transfected cells. As it has been previously described that endogenous PINK1 can be detected by western blot in HeLa cells (19), we decided to verify the editing efficacy of our CRISPR/Cas9 constructs in this cell line. As PINK1 is rapidly degraded in polarized mitochondria (20,21), cells were exposed to carbonyl cyanide 3-chlorophenyl hydrazone to induce mitochondrial membrane depolarization which promotes PINK1 stabilization and accumulation on the outer mitochondrial membrane. Figure 1B and C show PINK1 levels in scramble-transfected and *PINK1*-edited cells. The two gRNAs used induced 62 ± 6% and 34 ± 4% reduction, respectively, in protein levels compared to control cells. In contrast to PINK1, endogenous Parkin is not detectable in HeLa cells (22). Hence, we employed HEK-293T cells that have relatively high levels of endogenous protein (22,23). Figure 1E and F show that the two gRNAs designed induced a reduction in Parkin levels by 24 ± 9% and 51 ± 1%, respectively, compared to scramble-transfected samples.

### *PINK1* and *Parkin* deficiency induces changes in mitochondrial redox levels in SH-SY5Y cells

**Figure 2.**
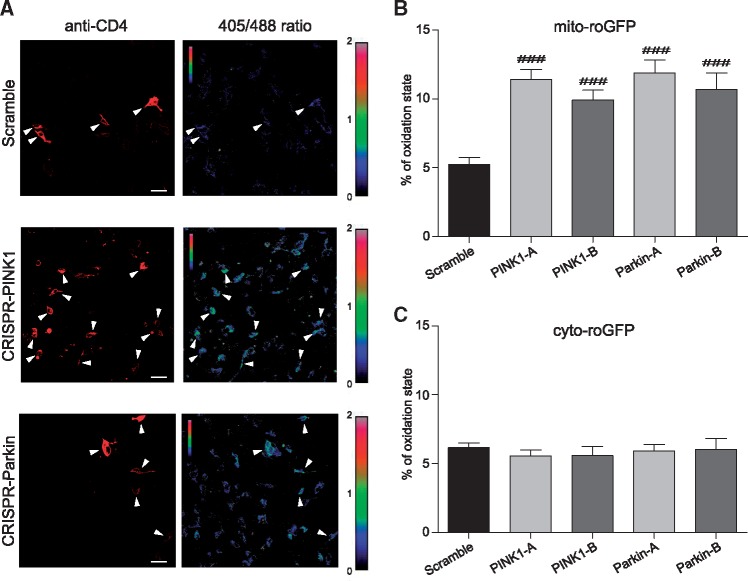
Analysis of the redox state in *PINK1*- and *Parkin*-edited SH-SY5Y cells—(**A**) Representative pseudocolor ratiometric images of scramble, CRISPR-PINK1 and CRISPR-Parkin transfected SH-SY5Y cells expressing the mito-roGFP probe. The 405/488 ratio was coded on a spectral color scale ranging from black (fully reduced) to red (fully oxidized), with the limits set after calibration. CD4 positive cells were identified after incubation with Alexa Fluor^®^ 647 anti-human CD4 Antibody (arrowheads in the figure). Scale bar: 30 µm. (**B**) Mitochondrial oxidation state of 60–70 CD4 positive cells from four independent experiments was measured using the mito-roGFP probe. (**C**) Cytosolic oxidation state of 40–50 CD4 positive cells from three independent experiments was measured using the cyto-roGFP probe. Fluorescence ratios (405/488) were calculated in scramble, CRISPR-PINK1 or CRISPR-Parkin transfected SH-SY5Y cells and normalized with respect with the ratios corresponding to the 100% reduced and oxidized state. Data are reported as mean ± SEM and statistical significance was determined by One way ANOVA and Dunnett’s *post-hoc* test. ^###^*P* < 0.001 compared to scramble.

Since *PINK1* and *Parkin* loss has been associated with increased generation of reactive oxygen species (ROS) and oxidative stress, we decided to test whether in our experimental conditions we were able to detect any redox changes at the mitochondrial and cytosolic level. To this aim we employed SH-SY5Y cells, rather than HeLa and HEK cells, because of their neuronal origin, catecholaminergic phenotype and their wide use in PD field (24). To monitor oxidative stress, a redox sensitive green fluorescent protein (roGFP2) reporter was used. This protein, which is characterized by the presence of two cysteine residues able to form a disulfide bond under oxidizing conditions altering its fluorescence properties (25), allows the measurement of cellular redox state in live cells, regardless of the absolute levels of probe concentration, through ratiometric imaging of 405 nm versus 488 nm excitation (25). To discriminate whether redox state alteration involves different cell compartments, we used two different variants of roGFP2: one was cytosolic (cyt-roGFP2), while the other one was specifically targeted to mitochondria (mt-roGFP2) as previously reported (25). After calibrating our system, as previously described (17), this technique was used to assess the cellular redox state in *PINK1*- and *Parkin*-targeted cells. In Figure 2A, representative pseudocolor ratio pictures of *PINK1*- and *Parkin*-deficient cells expressing mt-roGFP2 are reported. As represented in Figure 2B and C, the loss of either PINK1 or Parkin protein increased the oxidative state at the mitochondrial level while the cytosol redox potential remained unaffected.

### Superoxide dismutation rescues mitochondrial redox state alterations in *PINK1*- and *Parkin*-deficient SH-SY5Y cells

**Figure 3.**
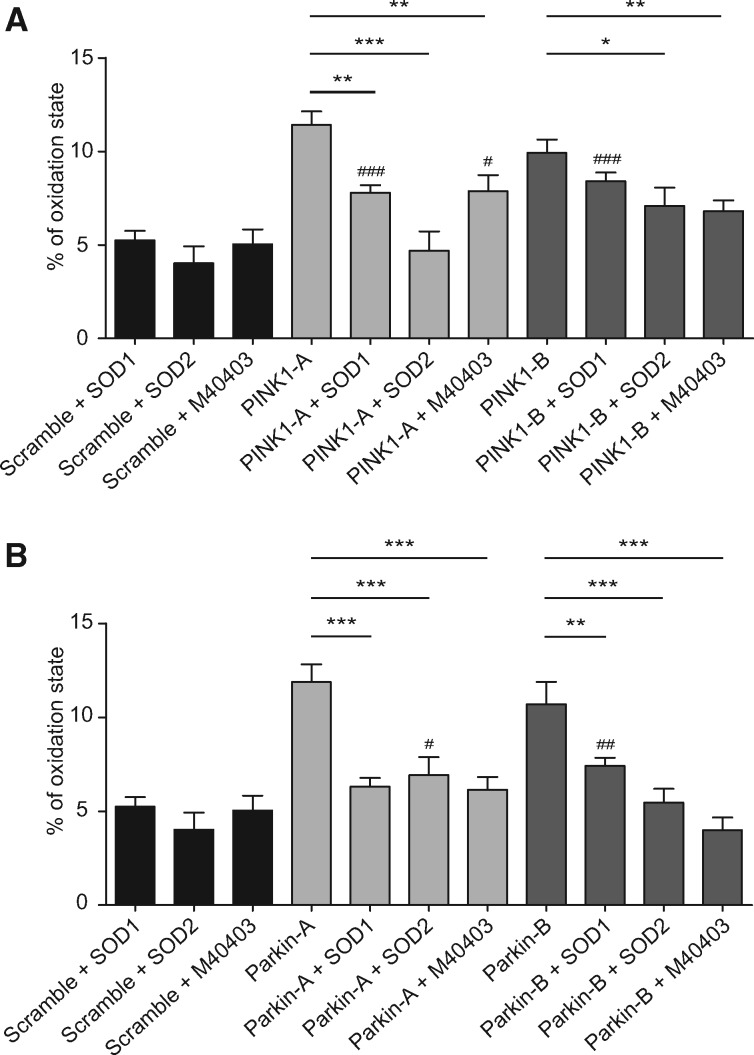
Effects of superoxide dismutation in the mitochondrial redox state of *PINK1*- and *Parkin*-edited SH-SY5Y cells—Wild-type, SOD1- or SOD2-overexpressing SH-SY5Y cells were transfected with scramble, CRISPR-PINK1- or CRISPR-Parkin vectors and treated with M40403 when indicated. Fluorescent ratios (405/488) were normalized with respect with the ratios corresponding to the 100% reduced and oxidized state. Data from three independent experiments are reported as mean ± SEM and statistical significance was determined by One-way ANOVA with Dunnett’s *post-hoc* analysis. ^#^*P* < 0.05, ^##^*P* < 0.01 and ^###^*P* < 0.001 compared with scramble transfected cells and *P < 0.05, **P < 0.01 and ***P < 0.001 as indicated in the graph.

To verify the contribution of superoxide radicals to the redox alteration observed in our cellular models of *PINK1* and *Parkin* deficiency, we analysed the redox state upon loss of *PINK1* or *Parkin* in previously described SH-SY5Y cells stably overexpressing SOD1 or SOD2 (17) by means of the roGFP2 probe. Since no alteration in the cytosolic redox state was observed, we evaluated the effects of overexpressing SOD1 or SOD2 solely on the mitochondrial oxidative state. The results obtained in *PINK1*-deficient cells (Fig. 3A) showed that the increased removal of superoxide radicals was able to reduce the oxidative state at the mitochondrial level, with SOD2 generally having a greater effect than SOD1. Similar results were also obtained in the *Parkin*-deficient cells. As shown in Figure 3B, the overexpression of either SOD1 or SOD2 was all able to reduce the mitochondrial oxidative state.

We next analysed the potential of the SOD mimetic compound M40403 to ameliorate *PINK1*/*Parkin*-related cell defects. Encouragingly, the administration of the SOD-mimetic drug M40403 significantly restored the redox state of both *PINK1* and *Parkin*-deficient cells (Fig. 3A and B). Overall, these results demonstrate that loss of PINK1 or Parkin protein leads to increased oxidative stress in mitochondria, which can be ameliorated by genetic or pharmacological upregulation of superoxide dismutation. We next sought to determine whether superoxide dismutation could also provide some improvements in mitochondria morphology in *PINK1*/*Parkin* loss of function models.

### *PINK1* and *Parkin* deficiency leads to changes in mitochondrial morphology in SH-SY5Y cells

**Figure 4.**
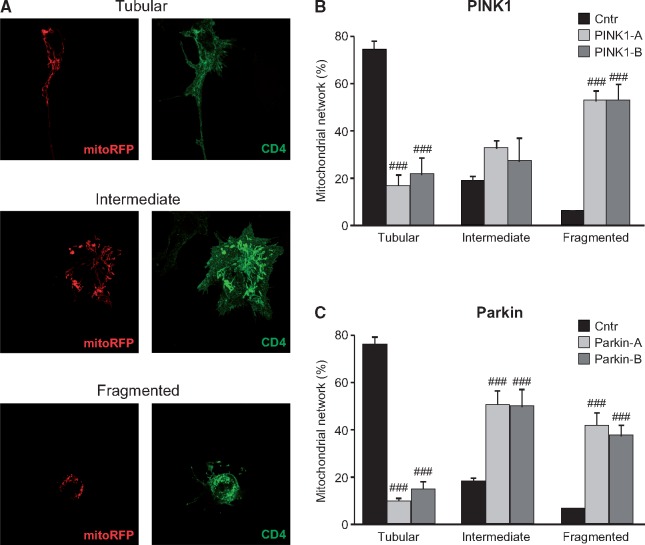
Mitochondrial network in *PINK1*- and *Parkin*-edited SH-SY5Y cells—SH-SY5Y cells were co-transfected with mitoRFP and CRISPR/Cas9 vectors. (**A**) Cells were stained using the anti-CD4 antibody conjugated to 488 fluorochrome and nuclei were counterstained using Hoechst. Mitochondrial network was scored as tubular, intermediate and fragmented. (**B** and **C**) The percentage of cells with a specific mitochondrial network was determined as a percentage of the total number of transfected cells counted. The data analysis was performed in a blind manner. Data are expressed as mean ± SEM of at least four independent experiments. Statistical significance was assessed by One-way ANOVA with Dunnett’s *post-hoc* analysis. ^###^*P* < 0.001 compared with scramble transfected cells.

In light of the mitochondrial redox state alteration detected in the previous experiments and since both mitochondrial ROS production and *PINK1/Parkin* deficiency have been described to induce mitochondrial fragmentation in several mammalian cell models (26–31), we next analysed mitochondrial morphology in CRISPR/Cas9 edited SH-SY5Y cells. Using the CD4 reporter to monitor the *PINK1*- and *Parkin*-targeted editing by anti-CD4 immunofluorescence, we evaluated the mitochondrial network in CD4^+^ cells. As previously reported, mitochondrial network was scored as tubular, which is characterized by elongated mitochondria with high interconnectivity, intermediate, which includes a mixture of circular and short tubular mitochondria, and fragmented, which is mainly constituted by very small and round mitochondria (17,32) (Fig. 4A). Importantly, to avoid unintentional bias, mitochondrial morphology was scored blind to the experimental conditions. Consistent with the aforementioned works, in comparison to control-transfected cells, *PINK1* deficiency led to alterations in mitochondrial morphology. Specifically, as shown in Figure 4B, a significant decrease in the percentage of mitochondria with tubular morphology was observed with a concomitant accumulation of organelles with intermediate and fragmented morphology. As expected, the loss of *Parkin* in our cell model also strongly decreased the number of tubular mitochondria while increasing the amount of intermediate and fragmented organelles (Fig. 4C), in agreement with previously published results (31). For all conditions, a selection of representative images are shown in Supplementary Material, Figures S1 and S2.

### Superoxide dismutation induces rescue of mitochondrial morphological alterations in *PINK1*- and *Parkin*-deficient SH-SY5Y cells

**Figure 5.**
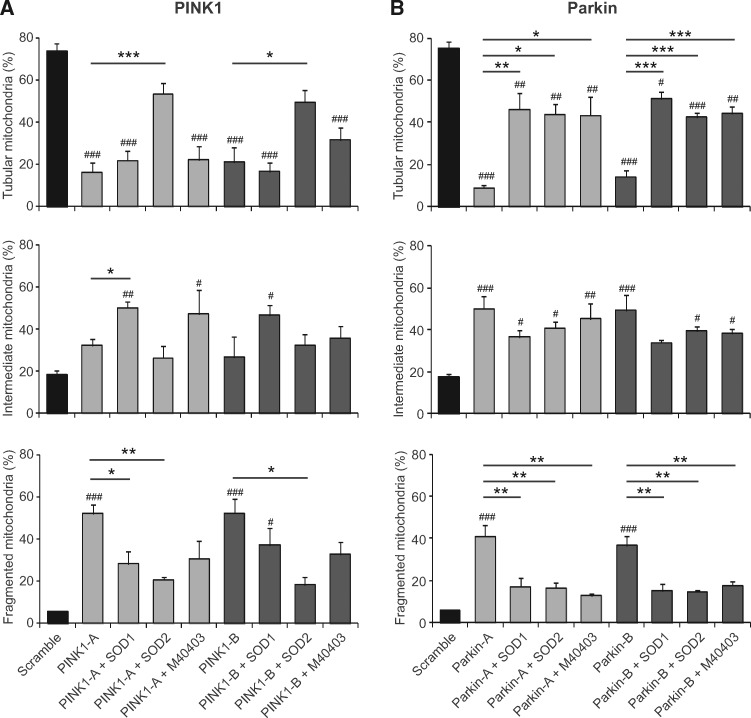
Effects of superoxide dismutation in mitochondrial morphology of *PINK1*- and *Parkin*-edited SH-SY5Y cells—Wild-type, SOD1- or SOD2-overexpressing SH-SY5Y cells were co-transfected with mitoRFP—and (**A**) CRISPR-PINK1-vectors or (**B**) CRISPR-Parkin-vectors and treated with M40403 when indicated. Mitochondrial network was scored as tubular, intermediate and fragmented. The percentage of cells with a specific mitochondrial network was determined as a percentage of the total number of CD4 positive cells counted. At least 35 CD4 positive cells were analysed per experiment. Data are expressed as mean ± SEM of at least three independent experiments. Statistical significance was assessed by One-way ANOVA with Dunnett’s *post-hoc* analysis. ^#^*P* < 0.05, ^##^*P* < 0.01 and ^###^*P* < 0.001 compared with scramble transfected cells and *P < 0.05, **P < 0.01 and ***P < 0.001 as indicated in the graphs.

The protective effects of superoxide dismutation were then analysed in SOD1- or SOD2-overexpressing SH-SY5Y cells. As reported in Figure 5a, both the enzymes were able to partially rescue the mitochondrial alterations induced by loss of PINK1 protein, but the effects were much more pronounced by the overexpression of SOD2. While SOD1 partially reduced mitochondria fragmentation by increasing the number of cells with an intermediate mitochondrial morphology, in the presence of SOD2 the reduction of fragmented organelles correlated with a restoring of tubular mitochondria. Similar to *PINK1-*edited cells, the mitochondria morphological impairment in *Parkin*-targeted SH-SY5Y cells was significantly rescued by the overexpression of either SOD1 or SOD2. In both conditions, the reduction of fragmented organelles correlated with a restoring of tubular mitochondria. Representative images are shown in Supplementary Material, Figures S1 and S2.

Having verified the beneficial effects of SOD enzymes we then evaluated the potential protective role of the M40403 SOD-mimetic compound. Interestingly, in *PINK1* deficient cells, we observed a protective trend of M40403 against mitochondria fragmentation with an increase in both the intermediate and the tubular states, although this did not reach statistical significance in these assays (Fig. 5A). However, the effects were greater in *Parkin* deficient cells: the treatment with M40403 significantly reduced the number of fragmented mitochondria while restoring tubular mitochondria (Fig. 5B).

### *Pink1* and *parkin* mutant *Drosophila* brains display elevated ROS levels

**Figure 6.**
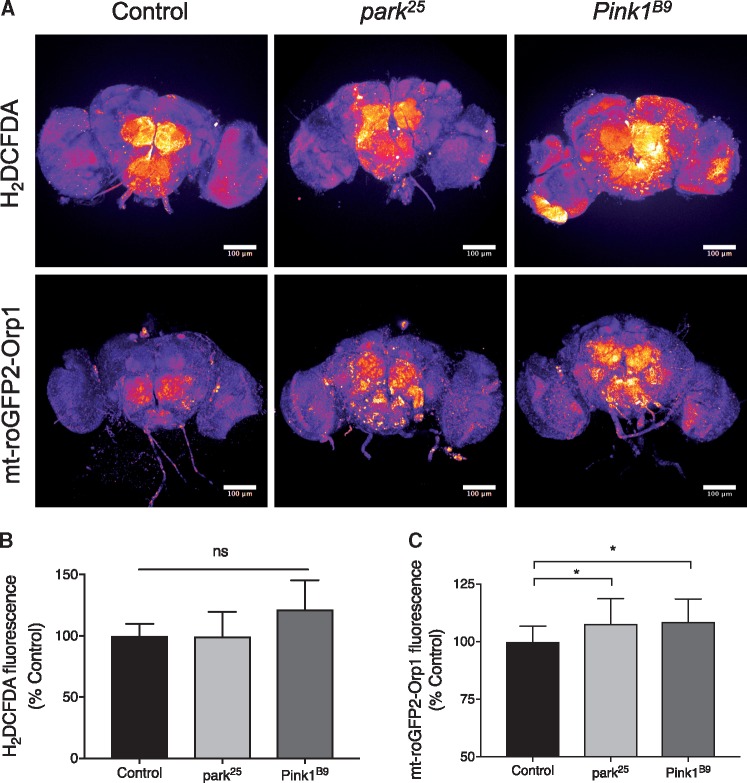
Analysis of redox state in *Pink1* and *parkin* deficient flies. (**A**) Pseudocolour representative images of adult *Drosophila* brains imaging cytosolic (H_2_DCFDA) or mitochondrial (mt-roGFP2-Orp1) ROS reporters in control (*w^1118^*), *park^25^* or *Pink1^B9^* adult brains. Scale bars = 100 µm. (**B**) Quantification of cytosolic (H_2_DCFDA) ROS normalised to control; control *n* = 10, *park^25^ n* = 9 and *Pink1^B9^ n* = 9. (**C**) Quantification of mitochondrial (mt-roGFP2-Orp1) ROS normalised to control; control *n* = 16, *park^25^ n* = 20 and *Pink1^B9^ n* = 16. Charts show mean ± SD. Statistical analysis used Kruskal–Wallis with Dunn’s multiple comparisons test (*P < 0.05; ns, non-significant).

Having demonstrated the potential for SODs to reverse phenotypes associated with loss of PINK1 and Parkin proteins, to extend our analysis *in vivo* we turned to well characterised *Drosophila* models of *PINK1/parkin* deficiency. *Drosophila Pink1* and *parkin* mutants display a number of characteristic phenotypes analogous to the disease condition including locomotor deficits and neurodegeneration (33,34). To determine the level of oxidative stress we used established methods to analyse cytosolic and mitochondrial ROS in *Pink1* and *parkin* mutants brains. Using the H_2_DCFDA dye we found no significant change in cytosolic ROS in *Pink1* or *parkin* mutants (Fig. 6A and B). In contrast, the mito-roGFP2-Orp1 mitochondrial ROS reporter revealed increased mitochondrial ROS in both *Pink1* and *parkin* mutants (Fig. 6A and C).

### Superoxide dismutation-induced rescue in *Pink1* and *parkin* knockout *Drosophila* models

**Figure 7.**
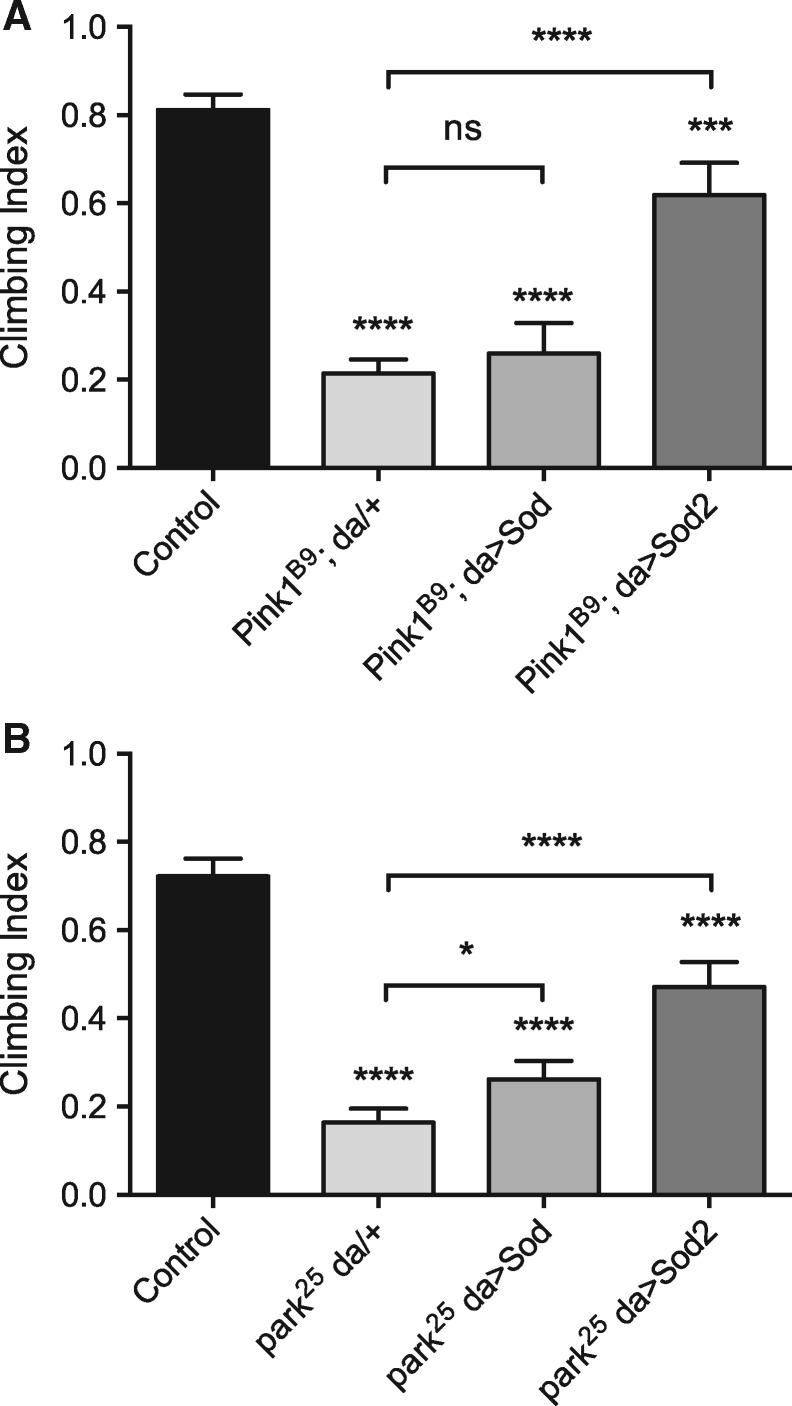
Analysis of *Sod* and *Sod2* expression in Pink1/parkin mutant locomotor assays. Climbing assay of (**A**) *Pink1* and (**B**) *parkin* mutant flies upon ubiquitous (*da-GAL4* driver) transgenic expression of *Sod* or *Sod2*. Charts show mean and 95% CI, *n* > 100 animals. Statistical analysis used Kruskal–Wallis with Dunn’s multiple comparisons test (*P < 0.05, ***P < 0.001, ****P < 0.0001; ns, non-significant).

To assess the contribution of ROS to *Pink1* and *parkin* mutant phenotypes, these mutants were genetically combined with transgenic lines expressing *Drosophila Sod* (homologous to mammalian *SOD1*) or *Sod2* (homologous to mammalian *SOD2*) and the ubiquitous *da-GAL4* driver, and assayed for locomotor ability (via negative geotaxis or climbing). In *Pink1* mutants *Sod* expression did not rescue climbing ability while *Sod2* substantially suppressed the climbing defect (Fig. 7A). Similarly, in *parkin* mutants expression of *Sod2* substantially rescued climbing ability; however, here *Sod* expression also modestly improved locomotion (Fig. 7B).

**Figure 8.**
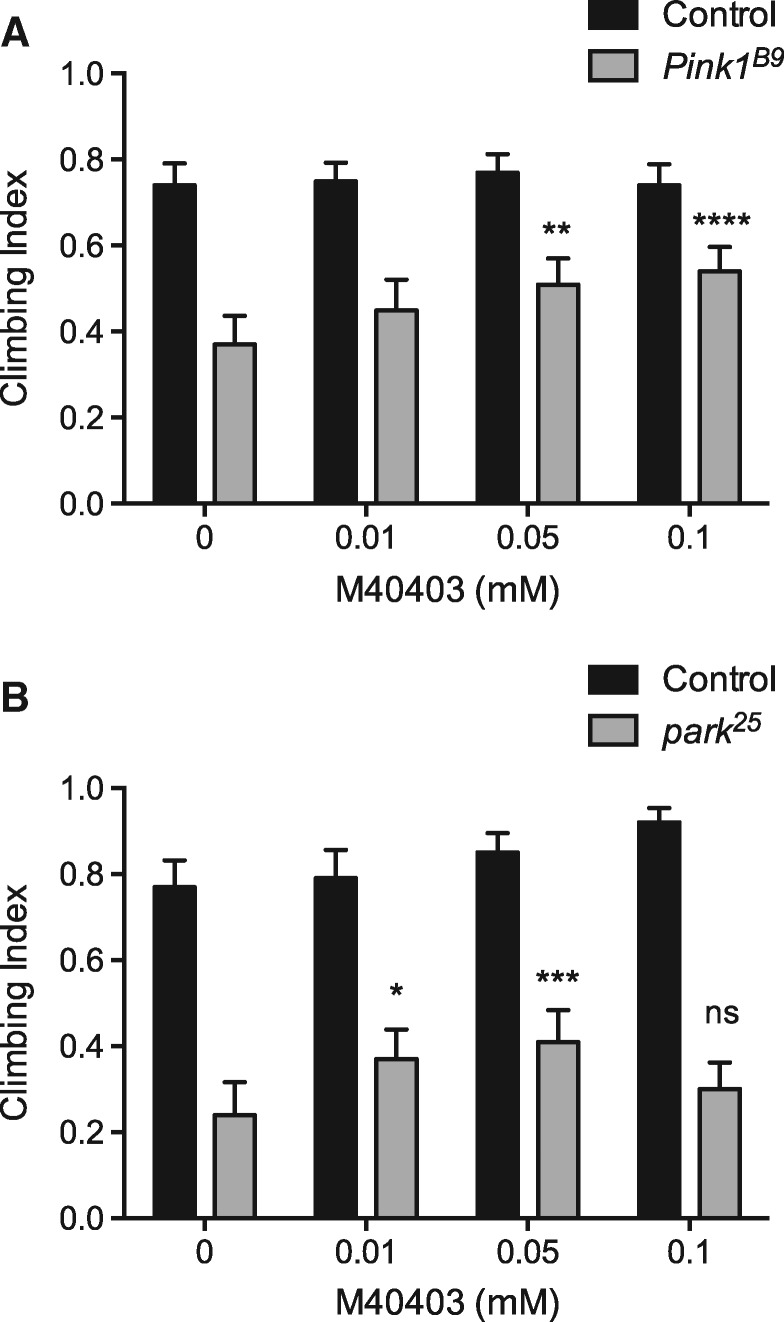
Effect of M40403 on *Pink1/parkin* mutant motor ability. Climbing assay of (**A**) *Pink1* and (**B**) *parkin* mutant flies treated or not with indicated concentrations of M40403. Charts show mean and 95% CI, *n* > 100 animals. Statistical analysis used two-way ANOVA with Tukey’s multiple comparisons test (*P < 0.05, **P < 0.01, ***P < 0.001, ****P < 0.0001; ns, non-significant).

We next wanted to address whether the M40403 SOD-mimetic could provide beneficial effects *in vivo*. *Pink1* and *parkin* mutant and control flies were raised on food dosed with varying concentrations of M40403 and climbing ability was again tested. In parallel, animals of the same genotypes were raised on standard non-drug treated food for comparison. For both *Pink1* and *parkin* mutants M40403 treatment significantly improved climbing ability (Fig. 8). The rescue was largely in a dose dependent manner within the range analysed. However, while in *parkin* mutants a modest rescue was seen with the lowest dose (0.01 mm), which was not significant in *Pink1* mutants, the beneficial effects were lost at the highest dose (0.1 mm), which afforded the best rescue in *Pink1* mutants. Taken together these results indicate that increasing endogenous or exogenous SOD activity can be beneficial in two *in vivo* models of PD.

## Discussion

Strong evidence exists linking oxidative stress and mitochondria dysfunction to both familial and sporadic PD cases (1). Accumulating indications also support that elevated ROS levels and abnormalities in mitochondria morphology are interconnected (3). As fragmented mitochondria are often observed in human pathological conditions, including PD (35), the reversal of such a morphological phenotype, through the modulation of endogenous antioxidant levels or by the application of exogenous antioxidants, might represent a valid therapeutic strategy. Nevertheless, clinical trials on PD patients based on antioxidant drugs have yielded mixed results (36). A possible explanation is that most of the tested molecules do not target the primary cause of the oxidative stress, i.e. excessive superoxide anion production, but rather the downstream effects. In cells, superoxide anions are mainly formed in mitochondria during oxidative ATP production, when a small leakage of electrons from the electron transport chain can directly react with oxygen to produce superoxide radicals (16). Accordingly, a treatment strategy for oxidative stress is likely to be more effective if it targets the origin of ROS generation.

In the present work we analysed the therapeutic potential of antioxidant molecules capable of decreasing superoxide radicals in two genetic models of PD, based on loss of PINK1 or Parkin function. We first established that the loss of PINK1 or Parkin protein in human neuroblastoma SH-SY5Y cells induces an increase in the mitochondrial oxidative state while the cytosolic redox state remains unaltered. Moreover, in both *PINK1*- and *Parkin*-deficient cells we observed a strong increase in the number of fragmented mitochondria, in comparison to wild-type cells. Interestingly, the overexpression of either SOD1 or SOD2 was able to prevent alteration in the redox state of mitochondria and to partially rescue the mitochondria fragmentation, albeit with more pronounced effects seen by SOD2 overexpression. Having demonstrated *in vitro* the beneficial effects of the superoxide dismutating endogenous enzymes, we then moved to a relevant *in vivo* model. Consistent with the *in vitro* findings, overexpression of Sod2, and to a lesser extent Sod, ameliorated locomotion defects in *Pink1* or *parkin* mutants.

Consistent with our results, it has been previously reported that in SH-SY5Y cells *PINK1* gene silencing increases the level of mitochondrial superoxide which, in turn, promotes mitochondrial fragmentation (27), and superoxide radicals have been proposed as essential mediators in triggering fragmentation of the mitochondrial network (27). In this frame, the protective effects observed by overexpressing SOD1, in terms of mitochondrial morphology and redox state, could be explained considering that, besides the cytosolic localization, the protein is also found in the mitochondrial intermembrane space.

In an important extension to the genetic manipulation of superoxide dismutation, we also showed that treatment with the SOD-mimetic compound M40403 reversed several effects of loss of PINK1/Parkin protein. In both *PINK1*- and *Parkin*-deficient cell models, M40403 preserved the redox state of these organelles and inhibited mitochondria fragmentation, with effects that were more pronounced in *Parkin* deficient cells. Similarly, we also observed beneficial effects from the systemic administration of M40403 in *Pink1* and *parkin* mutant flies. Interestingly, the dose-response to M40403 differed slightly between the two mutant models. In *Pink1* mutants increasing M40403 concentration provided an increasing benefit across the concentrations tested, becoming substantial in the highest dose. However, in *parkin* mutants, although there was a dose-dependent improvement in locomotion across low and mid-range concentrations, which was lost at the highest dose, the magnitude was appreciably less than in *Pink1* mutants. The reason for the slightly weaker rescue is not currently clear but in our experience *parkin* mutants display slightly stronger phenotypes than *Pink1* mutants, which is evident in the generally slightly poorer climbing performance seen here. Nevertheless, these results demonstrate that abrogating the damaging effects of superoxide is beneficial in these *in vivo* models of Parkinson’s disease.

While it is currently unclear exactly how loss of PINK1 or Parkin leads to the elevated oxidative stress, they are considered to play a central role in mitochondrial quality control, promoting mitophagy and other related processes [reviewed in (37,38)]. A breakdown in quality control processes will lead to accumulated dysfunctional mitochondria which will inevitably produce high levels of ROS. This provides a clear molecular basis for elevated oxidative stress in *PINK1/parkin* linked PD cases, but oxidative stress is also a feature of idiopathic PD pathology (39). In dopaminergic neurons oxidative stress has been suggested to arise at least in part from the oxidation of dopamine, generating both ROS and very reactive electron-deficient dopamine-quinones (DAQs) (40,41), which can conjugate a wide variety of macromolecules. Notably, Parkin as well as other PD-related factors such as DJ-1 and alpha-synuclein have been found to be modified by DAQs (42–44). Interestingly, SOD2 has also been described to be modified by DAQ affecting its activity and causing aggregation (45). Reducing this defence mechanism will inevitably lead to increases in oxidative stress. Moreover, very recently copper-deficient SOD1 aggregates as well as reduced enzymatic activity of SOD1 have been observed in vulnerable brain regions of idiopathic PD patients (46). Since SOD1 requires copper for catalysis, it is also notable that levels of copper and Copper transporter protein 1 are significantly reduced in remaining substantia nigra and locus coeruleus neurons of PD patients (47), suggesting that a reduction in cellular copper may affect the ability of vulnerable neurons to protect against oxidative stress.

In conclusion, all of this evidence supports a fundamental role for ROS derived oxidative stress as a contributing factor across the spectrum of PD. While further work in needed to understand the nature of detrimental effects of superoxide production and how this leads to neuronal cell death, our results presented here support the further exploration of endogenous and exogenous SOD-related molecules as a therapeutic strategy against PD.

## Materials and Methods

### Antibodies

The following antibodies were used: rabbit monoclonal anti-PINK1 antibody (D8G3, Cell Signalling Technology), mouse monoclonal anti-parkin antibody (sc-32282, Santa Cruz Biotechnology), rabbit Polyclonal anti-GAPDH antibody (Origene), anti-mouse and anti-rabbit IgG peroxidase antibodies (Sigma), Alexa Fluor^®^ 488 anti-human CD4 antibody (clone OKT4, BioLegend) and Alexa Fluor^®^ 647 anti-human CD4 antibody (clone OKT4, BioLegend).

### Guide RNAs cloning into the CRISPR/Cas9 vector

Endogenous *PINK1* and *parkin* genes have been edited by means of the CRISPR/Cas9 system. All guide RNA candidate target sequences (gRNAs) contain the NGG sequence at the 3’ end (PAM domain). The gRNAs were chosen to exhibit low off-target activity in genic regions of the human genome, according to CRISPR RGEN online tools (http://rgenome.net/cas-offinder/). The gRNAs were cloned in an expression vector containing a Cas9 nuclease expression cassette and a CD4 reporter [GeneArt^®^ CRISPR Nuclease (CD4 Enrichment) Vector Kit, Life Technologies] according to manufacturer’s instructions. Positive colonies were confirmed by DNA sequencing. For each gene of interest two gRNAs were selected to introduce double strand break within the genomic DNA. A scramble sequence was used as negative control. The following pairs of primers were used:

1) PINK1-A top5′-CCGCTTCTTCCGCCAGTCGGGTTTT-3′;

PINK1-A bottom5′-CCGACTGGCGGAAGAAGCGGCGGTG-3′;

2) PINK1-B top5′-CGTAATTCACATTGGAGCAGGTTTT-3′;

PINK1-B bottom5′-CTGCTCCAATGTGAATTACGCGGTG-3′;

3) PARKIN-A top5′-CAGTTGCGTGTGATTTTCGCGTTTT-3′;

PARKIN-A bottom5′-GCGAAAATCACACGCAACTGCGGTG-3′

4) PARKIN-B top5′-TCCACCCGAGTCAAGCTCTGGTTTT-3′

PARKIN-B bottom5′-CAGAGCTTGACTCGGGTGGACGGTG-3′

5) Scramble-top5′-GCACTACCAGAGCTAACTCAGTTTT-3′

Scramble-bottom5′-TGAGTTAGCTCTGGTAGTGCCGGTG-3′

### Cell culture and drug treatments

Human embryonic kidney cells (HEK293T, ATCC^®^) and human adenocarcinoma cells (HeLa, ATCC^®^) were cultured in Dulbecco’s modified Eagle’s medium (DMEM, Thermo Fisher) supplemented with 10% fetal bovine serum (Thermo Fisher) at 37°C and 5% CO_2_. To assess PINK1 protein levels, HeLa cells were treated with 4 µM MG132 (Sigma-Aldrich) and with 10 µM carbonyl cyanide 3-chlorophenylhydrazone (CCCP, Sigma-Aldrich) for 24 h and 4 h, respectively, at 37°C before cell lysis. Human neuroblastoma SH-SY5Y cells (IST, Genova, Italy) were cultured in a 1:1 mixture of Ham's F12 and Dulbecco Modified Eagle Medium (Life Technologies) supplemented with 10% fetal bovine serum, in a 5% CO_2_ humidified incubator at 37°C. Previously described SOD1 and SOD2 stably overexpressing SH-SY5Y cells were used (17). When required, SH-SY5Y cells were treated with 10 µM SOD mimetic drug M40403 for 24 h at 37°C.

### Isolation of CD4 positive cells

Twenty-four hours after seeding in 10 cm dishes, HEK293T and HeLa cells were transfected with 10 μg of DNA using polyethylenimine (PEI, Polysciences) as transfection vehicle with a PEI to DNA w/w ratio of 4/1 and 2/1, respectively. To increase the number of transfected cells in the cell cultures, CD4 positive cells were isolated before the evaluation of PINK1 and parkin protein levels. For this purpose, 72 h after seeding, cells were subjected to CD4 isolation with the Dynabeads^®^ CD4 Positive Isolation Kit (Life Technologies), according to manufacturer’s instructions. Isolated CD4 positive cells were then plated into six well plates for western blot analysis.

### Western blotting

To assess PINK1 and Parkin protein levels, transfected HEK293T and HeLa cells were lysed in 20 mm Tris–HCl buffer, pH 7.5, containing 150 mm NaCl, 1 mm EDTA, 1% Triton X-100, 2.5 mm sodium pyrophosphate, 1 mm beta-glycerophosphate, 1 mm Na_3_VO_4_, protease inhibitor cocktail (Sigma) and kept on ice for 30 min. Clarified lysates were obtained by centrifugation at 17 500*g* for 15 min at 4°C. The detergent-soluble supernatant fractions were quantified by BCA assay (Pierce Biotechnology). Proteins (50 µg) were separated by SDS-PAGE, transferred on PVDF membranes (Immobilion, Millipore) and subjected to western blot analysis using appropriate primary and secondary antibodies. Immunoreactive proteins were visualized using enhanced chemiluminescence (GE Healthcare). Densitometry was performed by using Image J Software and endogenous GAPDH protein was used as loading control.

### Mitochondrial morphology analysis

Wild-type, SOD1 or SOD2 overexpressing SH-SY5Y cells were plated on fibronectin-coated coverslips in 24-well plates (120 000 cells/well) and 24 h later co-transfected with 0.25 µg of mito-RFP vector (a gift from Prof. Luca Scorrano, University of Padua, Italy) and 0.75 µg of CRISPR/Cas9 vector using Lipofectamine (Life Technologies) as transfection reagent according to manufacturer’s instructions. After fixation with 4% paraformaldehyde, cells were stained using the anti-CD4 antibody conjugated to Alexa Fluor^®^ 488 fluorochrome. Nuclei were counterstained using 0.16 μm Hoechst 33258 (Life technologies). Images were acquired using a Leica 5000B epifluorescent microscope with the 100× oil objective (numeric aperture: 1.30). Data analysis was performed on CD4 positive cells, in a blind manner and reported as percentage of cells with tubular, intermediate or fragmented morphology.

### Cellular roGFP analysis

Wild-type, SOD1 or SOD2 overexpressing SH-SY5Y cells were plated on fibronectin-coated plates (µ-Slide 8 well, Ibidi) at a density of 70 000 cells/well. The next day cells were co-transfected with 100 ng of cyto- or mito-roGFP and 600 ng of CRISPR-Cas9 vector using Lipofectamine (Life Technologies) according to manufacturer’s instruction. To identify CD4 positive cells, 48 h after transfection cells were incubated with 1:200 Alexa Fluor^®^ 647 anti-human CD4 antibody in phenol-red free media. Images were collected with a Leica SP5 confocal microscope with 40× objective (oil immersion, numeric aperture: 1.25–0.75). Fluorescence was collected between 500 and 530 nm using 405 and 488 nm as excitation wavelengths. Prior the experiments, cyto- and mito-roGFP probes were calibrated in presence of 1 mm H_2_O_2_ (100% oxidized state) and 4 mm DTT (0% oxidized state). To avoid photobleaching and/or laser-induced oxidation during calibration, images were acquired every 2 min using a wide pinhole and a fast scanning (512 × 512 resolution). Fluorescence settings were kept constant across experiments and conditions. Raw data were exported to ImageJ software (http://rsb.info.nih.gov/ij/). Each cells in the field was selected as region of interest (ROI) and the mean intensity of each ROI was then measured after appropriate background correction. Three to four independent experiments were performed and in each one, a total number of 20–30 CD4 positive cells per condition were quantified and analysed.

### Drosophila strains and culture

Flies were raised under standard conditions at 25°C on food consisting of agar, cornmeal, molasses and yeast. *Pink1^B9^* mutants were a kind gift from J Chung (34), and the UAS-*mito-roGFP2-Orp1* was provided by A. Sanz (48). *park^25^* mutants have been described previously (33). The following strains were obtained from the Bloomington *Drosophila* Stock Center: *da-GAL4* (#5460), UAS-*Sod* (#33605) and UAS-*Sod2* (#24494).

### *In vivo* ROS measurement

In *Drosophila*, ROS analysis was performed as previously described (49). Dichlorofluorescein (H_2_DCFDA; Sigma, D399) was used to measure total cytosolic ROS levels in 3–5 days old adults. Brains were dissected in PBS then incubated in 30 μM H_2_DCFDA for 10 min before being washed three times with PBS and imaged immediately. Mitochondrial ROS imaging was performed using mito-roGFP2-Orp1 reporter lines, 3–5 days old adult fly brains were dissected in PBS and imaged by excitation at 488 nm (reduced) or 405 nm (oxidized), with emission recorded at 510 nm. Images were acquired using an LSM880 confocal microscope (Zeiss) equipped with a 20× 0.8 NA. The maximum intensity of projected z-stacks from imaged brains was quantified using ImageJ.

### Locomotion assay

The mobility of flies from each genotype was assessed using a counter-current apparatus in a negative geotaxis climbing assay. One to two-day-old flies were placed in an empty plastic vial (2.5 cm diameter), gently tapped to the bottom, and the number of flies crossing a line at 8 cm height within a time period of 10 s was scored. Each animal was tested five times. The number of male flies tested per genotype was *n* > 150. To investigate whether the M40403 compound could rescue the strong phenotype of *Pink1^B9^* and *park^25^* mutants, the treatment was performed throughout development (from egg-laying to adult eclosion) through the addition of varying concentrations of M40403 during food preparation.

### Statistical analysis

Cell data were analysed on Prism by one-way ANOVA with the Dunnett’s *post-hoc* test. Climbing data were analysed by Kruskal–Wallis non-parametric test with Dunn’s *post-hoc* test for multiple comparisons. The effect of M40403 on climbing was analysed by two-way ANOVA with Dunnett’s *post-hoc* test for multiple comparisons. *P* < 0.05 was considered significant.

## Supplementary Material

Supplementary Material is available at *HMG* online.

*Conflict of Interest statement*. None declared.

## Supplementary Material

Supplementary Figure S1

Supplementary Figure S2
