# Supplementary material for: Superoxide dismutating molecules rescue the toxic effects of PINK1 and parkin loss
Source: Hum Mol Genet. 2018 Feb 24;27(9):1618–29. doi: 10.1093/hmg/ddy069 (PMC5905640; doi:10.1093/hmg/ddy069)
Supplement: Supplementary Figure S1 [file ddy069_pink1-suppl.pdf]

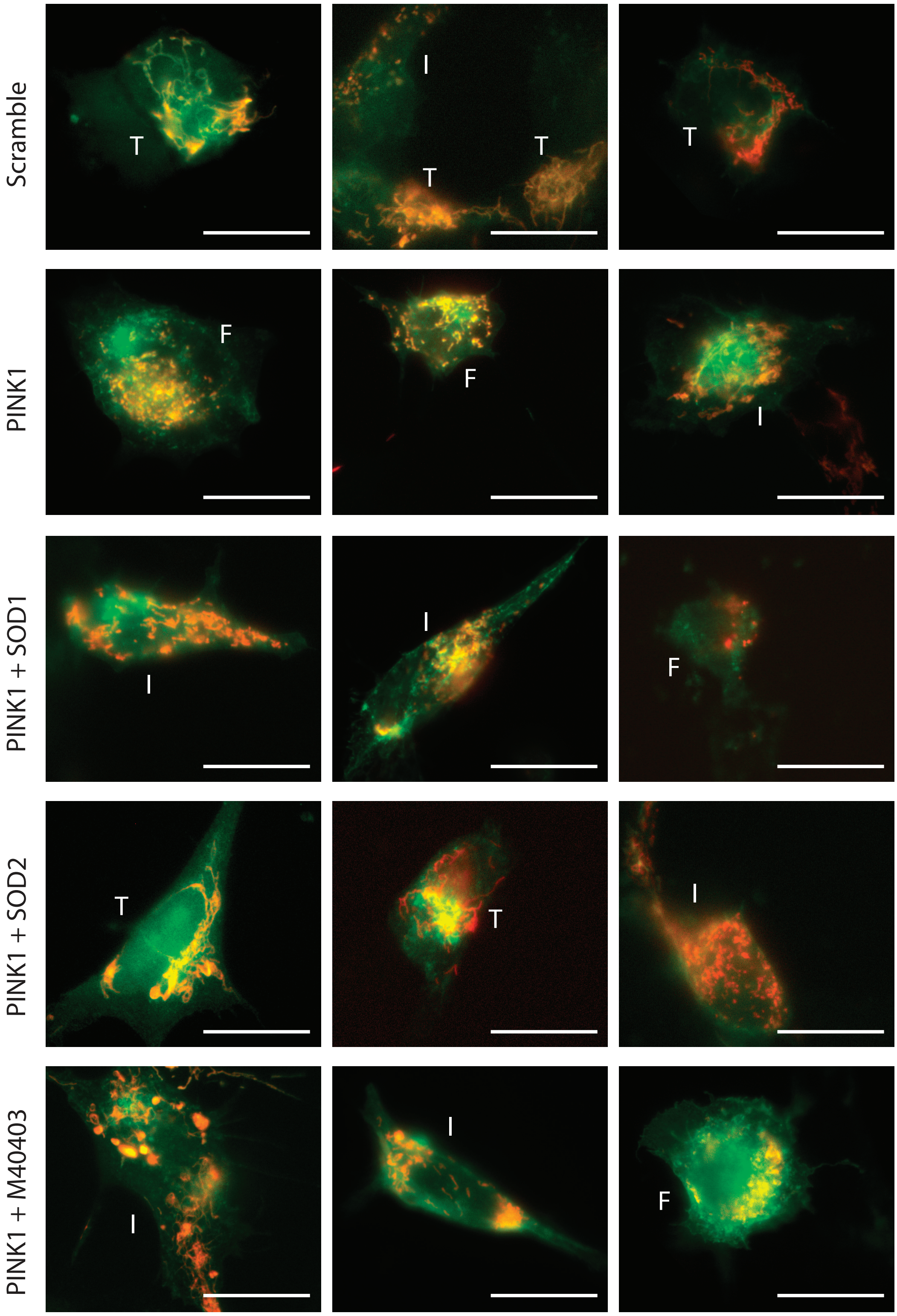

Supplementary Figure S1. Representative images of mitochondria morphology recorded in wild-type or SOD1 and SOD2 overexpressing SH-SY5Y cells transfected with scramble or CRISPR/Cas9-PINK1 vectors. Mitochondria are represented in red, the CD4 positive cells in green. Scale bar is 20 μm. T: tubular mitochondria; I: intermediate mitochondria; F: fragmented mitochondria
